# Palbociclib in combination with simvastatin induce severe rhabdomyolysis: a case report

**DOI:** 10.1186/s12883-019-1490-4

**Published:** 2019-10-22

**Authors:** Vardan Nersesjan, Klaus Hansen, Thomas Krag, Morten Duno, Tina D. Jeppesen

**Affiliations:** 1 Department of Neurology, Rigshospitalet, University of Copenhagen, Blegdamsvej 9, DK-2100 Copenhagen, Denmark; 2 Copenhagen Neuromuscular Center, Dep. of Neurology, Rigshospitalet, University of Copenhagen, Copenhagen, Denmark; 3 Department of Clinical Genetics, Rigshospitalet, University of Copenhagen, Copenhagen, Denmark

**Keywords:** Palbociclib, Simvastatin, Rhabdomyolysis, CYP3A4, *rs4149056*

## Abstract

**Background:**

Palbociclib is a selective well-tolerated antineoplastic drug used in the treatment of advanced HER2-negative, estrogen-receptor positive breast cancer that has shown significant improvement in progression-free survival. We present a patient that developed severe rhabdomyolysis with tetra-affection and loss of gait after initiating the first cycle of Palbociclib concomitantly with Simvastatin 40 mg treatment.

**Case presentation:**

A 71-year-old woman with metastatic breast cancer developed tetraparesis and near fatal rhabdomyolysis after initiation of first cycle Palbociclib. For 10 years prior to this treatment, the patient had been treated with Simvastatin without myalgia or other neuromuscular complaints prior to the first cycle of Palbociclib. The patient was admitted at the neurology department, where Palbociclib and Simvastatin were discontinued. The patient was aggressively hydrated and treated with intravenous immunoglobulin therapy with slowly remission and finally regaining independent gait function. Evaluation showed a negative myositis antibody work-up. Muscle magnetic resonance imaging showed edema in multiple foci, but skeletal muscle biopsy did not show necrosis. Post discharge genetic analysis showed single heterozygosity for nucleotide polymorphism *rs4149056*.

**Conclusion:**

We present a patient who developed severe rhabdomyolysis induced by a combination of Palbociclib and Simvastatin treatment. Rhabdomyolysis was most likely induced by toxic plasma concentrations of Simvastatin due to Palbociclibs inhibition of the CYP3A4 enzyme in combination with a decreased hepatic uptake of Simvastatin due to single nucleotide polymorphism *rs4149056*. The study underscores that combining Simvastatin and Palbociclib should be done cautiously and genetic testing of the rs4149056 SNP is warranted. If present, Simvastatin should be discontinued or replaced with a lesser myopathic statin in regard to patients risk of cardiovascular events.

## Background

Palbociclib is a selective antineoplastic drug for the treatment of advanced estrogen receptor–positive (ER), human epidermal growth factor receptor (HER) 2-negative breast cancer [1]. Since the FDA approval of Palbociclib in 2015 [2], more than 90.000 patients have been treated with Palbociclib [3]. Additionally two more cyclin-dependent kinase (CDK) inhibitors Ribociclib and Abemaciclib have reached FDA approval, and shown strong pre-clinical basis for the treatment of advanced ER positive breast cancer and potentially other solid tumors and hematological malignancies [4]. Palbociclib acts as a selective inhibitor of CDK4 and CDK6. Both are regulatory proteins in the cell-cycle enabling cell division, and inhibition of these proteins results in cell-cycle arrest and thus impairment of tumor growth [1].

**Fig. 1:**
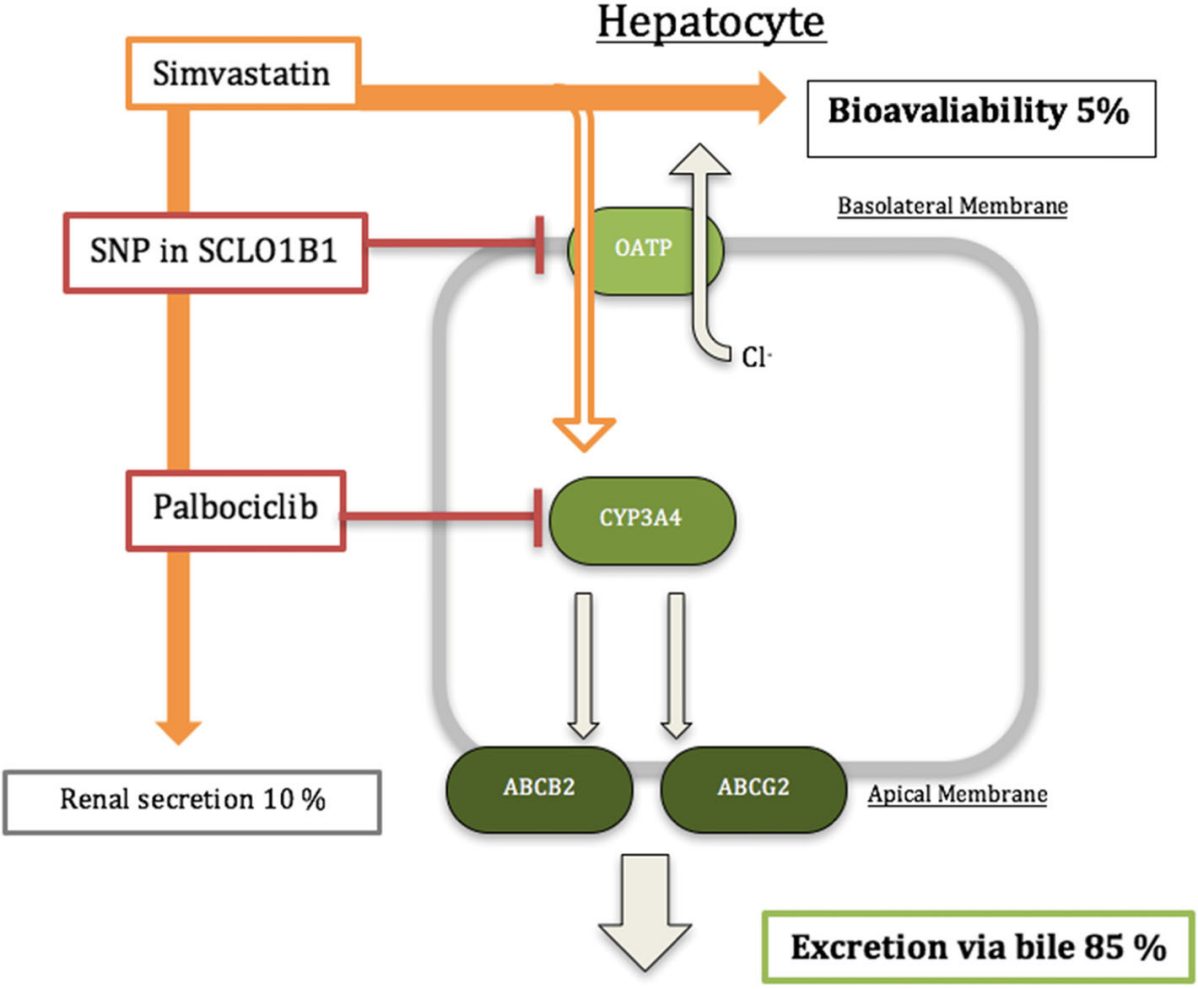
A simplified model of Simvastatin uptake into the hepatocyte. Simvastatin is transported by the organic-anion-transporting polypeptide (OATP) into the hepatocytes, metabolized by the CYP3A4 enzymatic system, and secreted via ATP dependent membrane transport efflux proteins ABCB2 and ABCG2 that pump foreign substances out of cells. Approx. 85% of Simvastatin is cleared via hepatocytes and bile excretion, 10% through renal clearance, and 5% of total drug concentration is available (termed bioavailability). The Single nucleotide polymorphism (SNP) *rs4149056* is a genetic mutation in the SLCO1B1 gene that codes for the OATP and when present results in a reduction in OATP activity, thus decreasing Simvastatin hepatocyte uptake and increasing bioavailability. Palbociclib is an antineoplastic drug used in HER2-negative, estrogen-receptor positive breast cancer, and acts as an inhibitor of the CYP3A4e enzyme

Palbociclib is considered to be well-tolerated and the most common adverse effects are neutropenia, leukopenia, fatigue and nausea [5]. However, a recent case-report presented a patient with a fatal outcome caused by kidney failure due to untreatable rhabdomyolysis, most likely induced the combination of Palbociclib and Simvastatin [6]. Simvastatin is cleared by first pass metabolism in hepatocytes by the CYP3A4 enzyme, and only 5% of the ingested drug is available after hepatic clearance [7]. Since Palbociclib is a time-dependent inhibitor of CYP3A4, the administration of Palbociclib may result in decreased Simvastatin clearance and elevated drug availability, possibly reaching toxic levels (see Fig. 1 ).

Myalgia, cramps and stiffness are side effects reported in 10% of patients treated with Simvastatin and is believed to be related to a subclinical myopathy/myositis, while significant rhabdomyolysis is rare [8]. The mechanisms of statin-related muscular adverse events are not completely understood, but it is believed that statins act either directly as a toxic metabolite in the myocyte, or indirectly by inducing antibodies against the HMG-CoA reductase resulting in an immune-mediated necrotizing myopathy [8]. The induction of rhabdomyolysis upon statin treatment seems to be dose-dependent [9]. Thus, Palbociclib-induced reduction of hepatocyte clearance may elevate plasma concentrations of statins and potentially result in rhabdomyolysis.

We present a 71-year-old woman on prior statin therapy that developed progressive proximal muscle weakness and fulminant rhabdomyolysis within days after 1 cycle of Palbociclib treatment.

## Case presentation

A 71-year-old woman presented with ten-day history of general weakness and muscle pain. The patient was on Simvastatin 40 mg daily for approximately 10 years without any complaints of side effects, especially no muscle pain or muscle fatigue.

In the year 2000, the patient was diagnosed with ER positive and HER-2 negative breast cancer. The patient underwent a surgical resection followed by adjuvant local radiation therapy of the pectoral region. In 2012 the patient had local recurrence of the tumor and was treated with a total mastectomy, followed by adjuvant treatment with aromatase inhibitors, which acts by facilitating key steps in the production of estrogen [10]. In 2017 the patient developed thoracic back pain and Magnetic Resonance Imaging (MRI) of the spine revealed four bone metastases in thoracic and lumbar vertebrae. A whole body positron emission tomography (PET) excluded additional metastases. 1 month later the patient was prescribed Palbociclib 125 mg daily for 21 days and Fulvestrant 500 mg on day one and 14 receiving 1 cycle of treatment. Prior to initiation of the first cycle of Palbociclib, the patient was in good condition with no complaints of myalgia, normal gait function, full bladder control and the light back-pain was managed with paracetamol and tramadol.

After 3 days of Palbociclib treatment the patient experienced onset of mild myalgia worsening over 7 days to pronounced muscle pain and severe proximal muscle weakness. After 10 days she could not raise her arms above shoulder level, she had difficulty getting up from a sitting position, and progressive impairment of walking that within 10 days resulted in complete loss of gait function and ability to stand upright without aid. Additionally, the patient experienced dark colored urine. There were no sensory complaints or sphincter dysfunction.

Neurological examination showed weakness of neck flexors and mainly proximal muscle weakness in both upper and lower limbs; shoulder abduction medical research council (MRC) grade 3, elbow flexion / extension grade 4, and bilateral hip flexion MRC grade 2. There was no independent gait function, and the patient was confined to a wheelchair. The patient could stand when assisted but could not walk. Cranial nerve examination, sensory evaluation and reflexes were unremarkable. The patient was initially admitted at a local emergency department under suspicion of metastatic spinal cord compression, but MRI of the spine showed, beside the known vertebral metastases, no sign of cord compression or myelopathy. Six days later the patient was transferred to our neurology department at a tertiary center for the suspicion of myositis. Simvastatin, Palbociclib, and Fulvestrant were immediately discontinued. At that time the patient had received Palbociclib daily for 21 days.

Blood samples showed increased creatine kinase (CK) 13,000 U/L on arrival at the neurology department increasing to above 22,000 U/L during day three to five reaching a plateau, and slowly decreasing to near normal on day 14. Plasma-myoglobin was elevated as well (10,400 μg/L) and decreased subsequently under treatment. Creatinine levels were low and glomerular filtration stayed within normal range throughout (Table 1). A diagnostic panel for detection of myositis-specific and myositis-associated autoantibodies was not contributive (Table 2).

**Table 1:** Plasma creatinine kinase, myoglobin, creatinine and eGFR during 10 days period of admission

	Day 1	Day 3	Day 5	Day 7	Day 10
Creatine kinase, U/L	13,000	> 22,000	> 22,000	4770	422
Myoglobin, μg/L	10,400	26,000	15,700	1790	216
Creatinine, μmol/L	60	47	44	42	35
eGFR, ml/min	90	> 90	> 90	> 90	> 90

Day 1, denotes the first day of admission to dep. of neurology

**Table 2:** Myositis specific and associated autoantibodies

Test name	Antibody	results
Myositis antibodies	52 kDa Ro Protein-IgG-P-Ab	Negative
	Isoleuc.-tRNA synthet-Ab (IgG)[OJ]	Negative
	Glycyl-tRNA synthet.-Ab (IgG) [Ej]	Negative
	Polymyositis(PL-12)-Ab (IgG)	Negative
	Polymyositis(PL-7)-Ab (IgG)	Negative
	Polymyositis(SRP)-Ab (IgG)	Negative
	Histidin-tRNA-ligase[Jo1]-Ab(IgG)	Negative
	EXOSC9-Ab (IgG) [PM-Scl75]	Negative
	EXOSC10-Ab (IgG) [PM-Scl100]	Negative
	Polymyositis(Ku)-Ab (IgG)	Negative
	SAE1-antistof(IgG)	Negative
	NXP2-Ab (IgG)	Negative
	MDA5-Ab (IgG)	Negative
	TIF1y-Ab (IgG)	Negative
	CHD-4 –Ab (IgG) [Mi-2]	Negative
	Mi-2a-Ab (IgG)	Negative
	chromodomain helicase DNA binding protein 4-Ab	Negative
HMG-CoA-reductase-IgG		< 3 units (negative)
ANA-IgG screening		negative

**Fig. 2:**
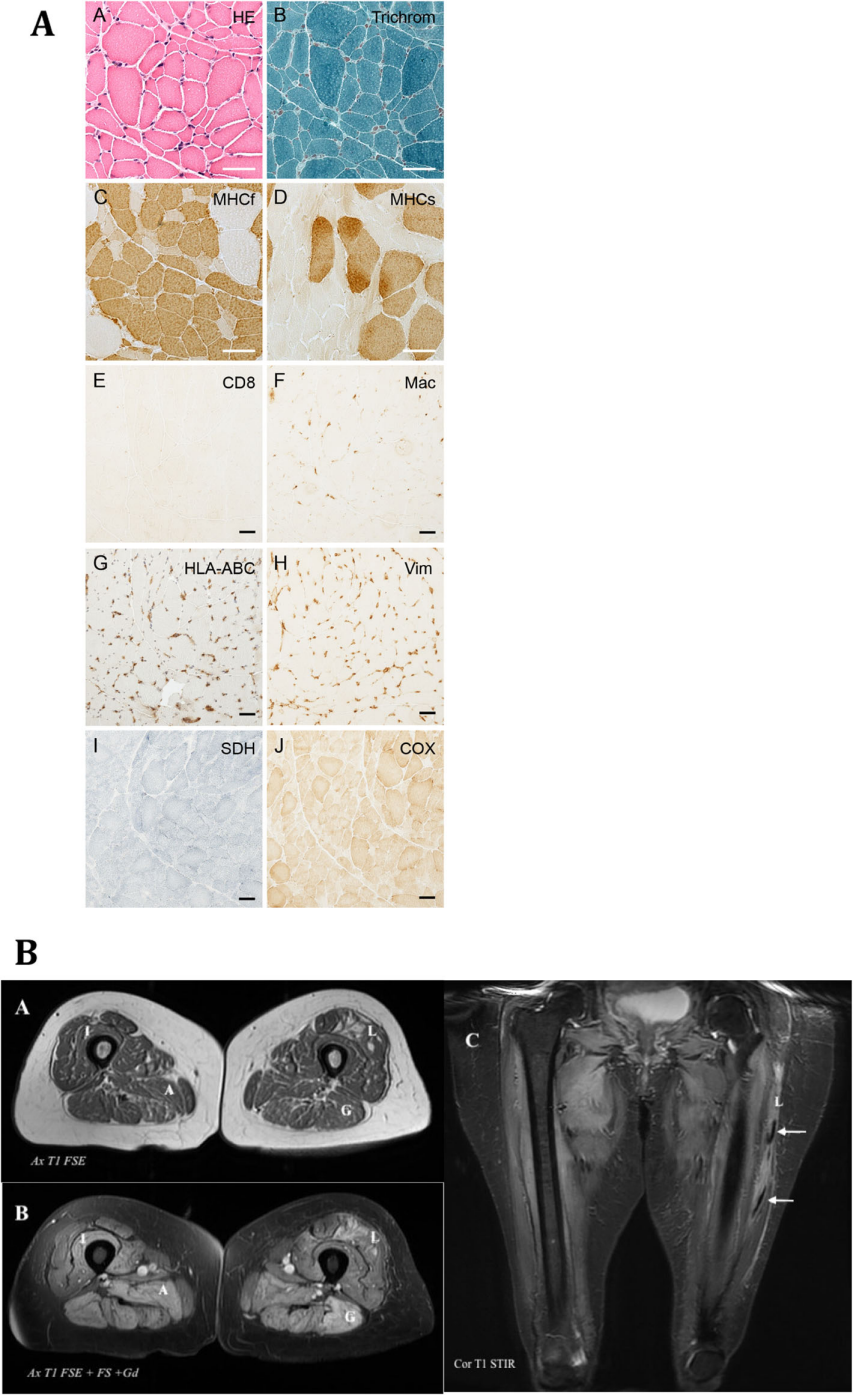
**A**: Skeletal muscle histopathology: A, Hematoxylin/Eosin (HE); B, Gomoris modified trichrom; C, fast myosin heavy chain (MHCf); D, slow myosin heavy chain (MHCs); E, CD8; F, macrophages (Mac); G, MHC Class 1 /HLA-ABC; H, Vimentin (Vim); I, succinyldehydrogenase SDH); J, Cytochrome C oxidase (COX). The black bar represents 50 μm. **B**: Magnetic resonance imaging of lower extremities. A, axial (Ax) T1-weighted Fast spin echo (FSE) sequence. B, Ax T1-wheigted FSE and fat saturated (Fs) with gadolinium contrast (Gd) sequence. C, coronal (Cor) T1-weighted Short-TI Inversion Recovery (STIR) sequence. In picture B the hyper intense signals in muscles Vastus Intermedius (I), Adductor Magnus (A), Vastus Lateralis (L) and Gracilis (G) indicate muscle edema. In picture C the arrows point to missing STIR signal In L that can be a sign of muscle necrosis

A muscle biopsy was obtained from the left vastus lateralis muscle. In hematoxylin and eosin (HE) stain the biopsy showed no signs of cell necrosis. No mononuclear cell infiltration verified in the macrophage and CD8+ stains, and no HLA-ABC positive fibers were found. There were no ragged-red fibers on trichrome staining and all fibers had normal cytochrome oxidase (COX) staining. There were no regenerated fibers in vimentin staining and biopsy showed type 2 atrophy (Fig. 2A).

An MRI of the lower extremities showed edema in multiple muscle groups (Fig. 2B). The right leg showed edema at the level of the adductor magnus, vastus intermedius, proximal vastus lateralis, distal vastus medialis and biceps femoris muscle. The left leg showed edema at the level of the vastus lateralis and biceps femoris. The MRI Short-TI Inversion Recovery (STIR) signal was abnormal at the left vastus lateralis indicating muscular necrosis (Fig. 2B).

To prevent kidney injury, the patient was hydrated with 4 l saline i.v., and a trial of intravenous immunoglobulin (IVIG) 0,4 g/kg over 5 days was initiated under suspicion of an autoimmune etiology for the muscle injury. This was initiated before autoantibody and muscle biopsy results were available.

The patient recovered gradually over 2 weeks. One month after hospitalization the patient had regained cane-assisted gait and was discharged to outpatient rehabilitation. Approximately one-year after hospitalization, the patient was seen at the neuromuscular outpatient clinic, showing no signs of neuro-muscular deficit.

A blood sample was obtained after discharge, and genetic analyses revealed heterozygous presence of the common SNP *rs4149056* in the *SLCO1B1* gene.

## Discussion

In the presented case, we describe a 71-year-old woman with prior non-problematic Simvastatin therapy who developed severe rhabdomyolysis after initiation of first cycle Palbociclib. The patient developed progressive proximal tetraparesis over a course of 10 days with loss of gait and standing function. She was admitted at our neurology department under suspicion of myositis. Due to fulminant rhabdomyolysis she was treated with hydration. The patient slowly recovered, regained gait function after 2 weeks of hospitalisation and after 1 year follow-up did not show any signs of neuromuscular deficiency.

We hypothesize that the addition of Palbociclib to Simvastatin treatment may have induced significant increase, and thus toxic plasma concentrations of Simvastatin in our patient, resulting in Simvastatin-induced rhabdomyolysis. Approximately 10% of patients treated with Simvastatin experience myalgia/myopathic problems while rhabdomyolysis is rather rare [11]. Simvastatin-induced adverse events are thought to be induced in a dose-dependent manner [9]. It is also shown that in statin-induced rhabdomyolysis, there is in 2/3 of cases concomitant treatment with a drug that inhibits CYP3A4. This underscores the high prevalence of rhabdomyolysis when statins and drugs that inhibit CYP3A4 are combined [11].

Palbociclib is a time-dependent CYP3A4 inhibitor as shown in clinical drug-to-drug interaction studies [12]. Simvastatin uptake is facilitated by the *SLCO1B1* gene product organic-anion-transporting polypeptide (OATP1B1) into the hepatocytes. Inside the hepatocytes, Simvastatin is metabolized by CYP3A4 enzymes and excreted out of the hepatocytes by membrane ATP-dependent efflux proteins (e.g. P-glycoprotein [P-gp / ABCB1], breast cancer resistance protein [BCRP / ABCG2]) into the bile, while only 5% ingested Simvastatin is actively bioavailable in the body [7, 8] (see Fig. 1 ). Genetic and pharmacokinetic studies have shown that the *rs4149056* SNP (both homozygous and heterozygous) is associated with reduced OATP1B1 activity, and thus increased Simvastatin concentrations that are significantly associated with myopathy [13]. The *rs4149056* SNP has been well studied and is present in approx. 15% of Caucasians [13]. In a population of rhabdomyolysis cases, the *rs4149056* SNP is shown to be as frequent as 25%, compared to 14% in a control population [14]. Thus, Simvastatin turnover may be reduced per se in patients with *rs4149056* SNP and these individuals may be prone to develop rhabdomyolysis compared to individuals which are homozygous for the reference allele. The severe rhabdomyolysis seen in our patient, may therefore be a result of increased Simvastatin plasma concentration induced by *rs4149056* SNP reduction of OATP1B1 activity and CYP3A4 enzyme inhibition by Palbociclib.

The patient was also treated with Fulvestrant which is an ER antagonist [15]. Fulvestrant has been associated with myalgia and elevated transaminases have been reported as well. Thus, it could be argued that Fulvestrant could be a potential mediator of the rhabdomyolysis. However, even though this has not been tested, we believe that rhabdomyolysis is more likely mediated by the combined treatment of Palbociclib with Simvastatin. Pharmacokinetic studies have shown that Fulvestrant does not have a significant interference with P450-mediated metabolism or affection of CYP3A4 activity [15], which in contrast, Palbociclib has.

In the present study, plasma concentrations of Simvastatin was not measured. Thus, the potential Palbociclib-induced elevated concentration of Simvastatin is merely a hypothesis. Some of the risk factors of developing statin-induced myopathy are older age and female gender [8]. Thus, it could be argued that these risk factors in itself have caused rhabdomyolysis in the patient, and the course of the rhabdomyolysis could have been milder in a younger patient. However, the direct time-line between initiation of Palbociclib treatment and rapid development of progressive, painful and proximal weakness with loss of gait function over a ten-day period strongly suggests an external mediator of statin-related rhabdomyolysis, which in or opinion is most likely to be mediated by Palbociclib. This hypothesis is substantiated by the fact that the patient was on Simvastatin treatment for 10 years with no previous muscular complaints.

This case is the second case that presents a potential statin-induced rhabdomyolysis after initiation of Palbociclib treatment. The first case was described previously by Nelson et al. [6]. The authors also found negative myositis autoantibody work-up and no other obvious cause to the severe rhabdomyolysis developed in direct relation to initiation of Palbociclib in a statin-treated patient. The patient presented died despite termination of Palbociclib, hydration and prednisolone treatment. The differences in clinical outcome may be due to differences in time-course of initiation of hydration and pre-morbid kidney function. The two cases indicate that terminating Simvastatin treatment in patients facing Palbociclib treatment should be considered. The progression free survival during Palbociclib treatment in first- and second-line therapy is 24 and 9 months, respectively and if cessation of Simvastatin is considered, it should persist until termination of Palbociclib [16]. Replacing Palbociclib with Ribociclib is not warranted since Ribociclib is a stronger inhibitor of the CYP3A4 enzyme [16]. Risk of cardiovascular events have to be taken into consideration and in patients with a high-risk profile, a dose reduction or ultimately a change to a lesser myopathic statin such as Rosuvastatin is recommended. Thus, if Simvastatin is not terminated, at least routine measurement of CK should be considered. Screening for the common *rs4149056* SNP could also be a reasonable approach when considering whether to discontinue Simvastatin or not in patients facing Palbociclib treatment. There is a report of a third case where a patient, who was treated with Palbociclib and Simvastatin, experienced elevated CK in a phase I trial, but unfortunately there is no additional work-up of this patient to suggest if Palbociclib is the mediator of the myopathy [17].

## Conclusion

We present the second case of severe rhabdomyolysis induced by a combination of Palbociclib and Simvastatin treatment. The reported case of rhabdomyolysis was most likely induced by Palbociclib-inhibition of the CYP3A4 enzyme, resulting in increased toxic levels of plasma concentration of Simvastatin. Additionally, our patient might have been more prone to develop rhabdomyolysis, due to the *rs4149056* single nucleotide polymorphism The study underscores that combining Simvastatin and Palbociclib should be done cautiously, and genetic testing is warranted to detect if the *rs4149056* SNP is present when treated with the aforementioned combination. If present, Simvastatin should be discontinued.

## Data Availability

Single subject data was generated through national electronic medical records, and original data of genetic testing, lab results, MRI and antibody panels are available upon request.

## Abbreviations

CDK: Cyclin dependent kinase
CK: Creatine kinase
ER: Estrogen receptor-positive
HER: Human epidermal growth factor receptor
IVIG: Intravenous immunoglobulin
MRC: Medical research council
MRI: Magnetic resonance imaging
OATP: Organic anion transporting polypeptide
PET: Positron emission tomography
SNP: Single nucleotid polymorphism
STIR: Short-TI inversion recovery
